# Chronic hepatitis B virus infection and occurrence of hepatocellular carcinoma in tree shrews (*Tupaia belangeri chinensis*)

**DOI:** 10.1186/s12985-015-0256-x

**Published:** 2015-02-13

**Authors:** Chun Yang, Ping Ruan, Chao Ou, Jianjia Su, Ji Cao, Chengpiao Luo, Yanping Tang, Qi Wang, Hong Qin, Wen Sun, Yuan Li

**Affiliations:** Department of Experimental Pathology, Guangxi Cancer Institute (Guangxi Tumor Hospital), Nanning, 530021 China; Department of Pathology, Guangxi Ruikang Hospital, Guangxi University of Chinese Medicine, Nanning, 530011 China

**Keywords:** Tree shrew (*Tupaia*), Hepatitis B virus, Hepatocellular carcinoma, Animal model

## Abstract

**Background:**

Hepatitis B virus (HBV) infection has been believed as a major cause of hepatocellular carcinoma (HCC) for a long time, however, the evidences of which are mostly from clinical and epidemiological investigations while there is no evidence from animal experiments. Tree shrew (*Tupaia*) is a small animal closely related to primates evolutionarily, with about 8 years of lifespan. Our previous study proved that tree shrews can be chronically HBV-infected after being inoculated neonatally with HBV. The present study reports the further results from the longer-term observation of these animals.

**Methods:**

Neonatal tree shrews were inoculated with sera from HBV-infected patient or tree shrew. Their serum samples and liver biopsies were collected periodically for detection of HBV markers as well as for histopathological and immunohistochemical examinations. Group A consisted of six tree shrews with chronic HBV-infection, and group B consisted of nine tree shrews without chronic HBV infection.

**Results:**

Periodical examinations on serum and liver biopsies of the animals in group A showed the progress of HBV infection, and two cases of HCC occurred at their late stage of life. The courses of HBV infection and the hepatic histopathological and immunohistochemical changes in the tree shrews were similar to those in humans. In contrast, neither HCC nor obvious hepatitis histopathological change was found among the tree shrews in group B.

**Conclusions:**

The course of HBV infection and the features of HCC discovered in tree shrews are similar to those of chronically HBV-infected humans. The tree shrew model might be used to investigate the underlying mechanisms favoring susceptibility for chronic HBV infection and disease progression.

## Background

Etiologically, the intimate relationship between human hepatitis B virus (HBV) infection and hepatocellular carcinoma (HCC), one of the most aggressive malignancies worldwide, is well recognized. The evidences of which, however, are mostly from clinical and epidemiological investigations [1-3] but not from systematic experimental studies. The main obstacle herein is the lack of suitable animal models, because the common laboratory animals are unsusceptible to HBV infection except chimpanzees.

The tree shrew (*Tupaia*) is a small animal classified currently in the order *Scandentia* and the superorder *Euarchonta*, with about 8 years lifespan. Compared to rats, mice, woodchucks and other laboratory animals, tree shrews show greater similarities with humans in many aspects such as genomic and immunological characteristics. Tree shrews, therefore, have been used in biomedical research for decades, especially in studies related to certain human viral diseases in recent years [4-9]. Since the early 1980s, studies have shown that tree shrews can be infected with HBV in vitro and probably in vivo [10-12], and HCC can be experimentally induced in tree shrews by aflatoxin B1 (AFB1) alone or in combination with HBV [13-15]. However, only one case of HBV alone-associated HCC has been reported so far, which was discovered in a tree shrew that had been originally captured in the wild during adulthood and then inoculated with HBV [14].

By study on a colony of laboratory-reared tree shrews that were inoculated with HBV at birth, we reported previously that six tree shrews were chronically HBV-infected, according to the criterion that HBV infection lasts longer than 48 weeks after inoculation [16]. Subsequently, we described that the histopathological changes in the liver of these persistently HBV-infected tree shrews were quite similar to those found in humans [17]. By continuous observation, HCC were discovered recently in two of the six chronically HBV-infected tree shrews but none among the nine controls that were confirmed as non-chronic HBV infection. This study investigated the progress from chronic HBV infection to the development of HCC in tree shrews.

## Results

### General information of the animals

All tree shrews observed in this study were from our previous investigations in which they were all inoculated with HBV at birth [16]. Group A consisted of six tree shrews that were chronically HBV-infected, while group B consisted of nine tree shrews that were confirmed as non-chronically HBV-infected. The average periods of observation on the animals in group A and B were 245.7 ± 90.9 and 249.1 ± 33.4 weeks after HBV-inoculation, respectively, and the difference of which was not statistically significant. Table 1 shows the general information of the animals in the two groups.

**Table 1:** **General information of the animals in group A and B**

**Group**	**Animal**	**Sex**	**Period of observation (week)** ^**a**^	**Condition at the end of study** ^**b**^
**A**	90-1	F	323	Sacrificed for a suspected liver tumor which was confirmed as liver cell dysplasia microscopically*.
	97-1	M	88	Natural death.
	98-2	F	319	Sacrificed because of HCC.
	121-1	M	278	Sacrificed because of HCC.
	122-1	M	190	Natural death.
	123-3	M	276	Alive.
	Mean ± SD		245.67 ± 90.86^c^	
**B**	108-4	F	311	Alive.
	117	F	206	Natural death.
	122-2	F	280	Alive.
	131-3	F	262	Alive.
	133-2	F	246	Natural death.
	133-3	F	260	Alive.
	136-2	M	241	Natural death.
	137-2	F	220	Alive.
	140-3	F	216	Alive.
	Mean ± SD		249.11 ± 33.44^c^	

^a^Period of observation, equals to the age of animal because all animals in this study were inoculated at birth and observed since then.

^b^Natural death, the death was without clear cause.

^c^The difference of observation period between two groups was not statistically significant (*P* = 0.607, Wilcoxon W = 67).

*Reference: Ruan P, Yang C, Su J, Cao J, Ou C, Luo C, Tang Y, Wang Q, Yang F, Shi J, et al.: Histopathological changes in the liver of tree shrew (Tupaia belangeri chinensis) persistently infected with hepatitis B virus. Virol J 2013, 10:333.

### Processes of HBV infection

**Figure 1:**
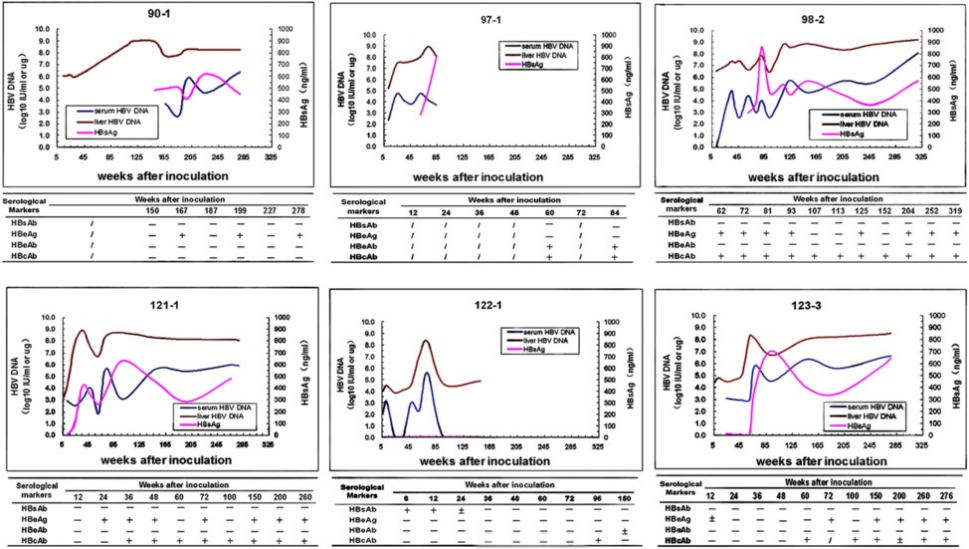
**Progress of HBV infection in tree shrews of group A.** The number on the top of each panel refers the name of animal. HBV DNA in serum and liver samples was detected by FQ-PCR. The result of HBV DNA detection was expressed as IU/ml in serum and as IU/μg liver DNA in liver. Serum HBsAg was detected by TRFIA and the result was expressed as ng/ml. Serum HBsAb, HBeAg, HBeAb and HBcAb were detected by ELISA and the results were expressed as “+” (positive), “-” (negative) or “±” (between positive and negative).

Figure 1 displays the time course of the HBV-infection markers in all six tree shrews of group A. Generally, the elevated levels of HBsAg in serum as well as HBV DNA in serum and in liver appeared shortly after HBV inoculation, followed by gradual increase with intermittent fluctuations. Typically, the serum HBeAg and HBcAb were positive at almost all time points in some animals such as 98–2 and 121–1, while HBsAb and HBeAb remained negative throughout.

On the other hand, as shown in Table 2, the HBV-infection markers of all tree shrews in group B indicate that none of them were chronically HBV-infected.

**Table 2:** **Serum HBV-infection markers in the tree shrews of group B**

**Animal**	**Infection markers in serum** ^*^	**Weeks after inoculation**						
		**6**	**12**	**24**	**36**	**48**	**~150** ^**^	**~250** ^**^
**108-4**	HBV DNA	/^**#**^	-	-	±	±	-	-
	HBsAg	/	/	/	/	-	-	-
	HBsAb	/	/	/	/	+	±	-
	HBeAg	/	/	/	/	-	-	-
	HBeAb	/	/	/	/	-	-	-
	HBcAb	/	/	/	/	-	-	-
**117**	HBV DNA	1.10 × 10^3^	-	±	-	-	1.92 × 10^3^	-
	HBsAg	±	±	-	-	-	-	-
	HBsAb	+	+	-	±	-	+	+
	HBeAg	-	-	-	-	-	-	-
	HBeAb	-	-	-	-	-	-	-
	HBcAb	-	-	-	-	-	+	-
**122-2**	HBV DNA	-	±	-	-	-	-	-
	HBsAg	±	±	-	-	-	-	-
	HBsAb	+	+	±	±	-	+	+
	HBeAg	-	-	-	-	-	-	-
	HBeAb	-	-	-	-	-	-	+
	HBcAb	-	-	-	-	-	-	+
**131-3**	HBV DNA	±	±	-	-	±	-	-
	HBsAg	-	-	-	-	-	-	-
	HBsAb	+	+	±	±	-	-	-
	HBeAg	-	-	-	-	-	-	-
	HBeAb	-	+	±	±	-	-	-
	HBcAb	-	+	-	±	-	-	+
**133-2**	HBV DNA	1.24 × 10^3^	3.01 × 10^3^	±	3.5 × 10^3^	-	-	-
	HBsAg	-	-	-	-	-	-	-
	HBsAb	+	+	±	-	-	-	-
	HBeAg	-	-	-	-	-	-	-
	HBeAb	+	+	±	-	-	-	-
	HBcAb	-	+	-	-	-	-	-
**133-3**	HBV DNA	±	1.04 × 10^3^	±	1.12 × 10^4^	1.46 × 10^4^	-	-
	HBsAg	-	-	-	-	-	-	-
	HBsAb	-	±	±	-	-	-	-
	HBeAg	-	-	-	-	-	-	-
	HBeAb	-	-	+	+	+	-	-
	HBcAb	-	+	+	+	+	-	-
**136-2**	HBV DNA	-	-	-	-	-	-	-
	HBsAg	-	-	-	-	-	-	-
	HBsAb	-	±	+	+	±	+	±
	HBeAg	-	-	-	-	-	-	-
	HBeAb	-	+	-	-	+	-	-
	HBcAb	-	+	-	+	+	-	-
**137-2**	HBV DNA	±	-	-	±	±	-	-
	HBsAg	-	-	-		-	-	-
	HBsAb	-	-	+	-	+	+	+
	HBeAg	-	-	-	+	-	-	-
	HBeAb	-	-	+	-	-	-	-
	HBcAb	-	-	-	-	-	-	-
**140-3**	HBV DNA	1.31 × 10^3^	±	-	/	-	-	-
	HBsAg	-	-	-	/	-	-	-
	HBsAb	-	+	-	/	-	±	-
	HBeAg	-	-	-	/	-	-	-
	HBeAb	-	-	-	/	-	-	+
	HBcAb	+	+	-	/	-	-	-

*HBV DNA in serum was detected by FQ-PCR, the results were expressed as values of IU/ml (positive), “-” (values < 0.5 × 10^2^ IU/ml) or “±” (values between 0.5 × 10^2^-1.0 × 10^3^ IU/ml). Serum HBsAg, HBsAb, HBeAg, HBeAb and HBcAb were detected by ELISA, the results were expressed as “+” (positive), “-” (negative) or “±” (between positive and negative).

**“~150” means around 150 weeks. “~250” means around 250 weeks, the last time of detection.

^#^Not tested at this time point.

### Occurrence of HCC

At the end of this study, two cases of liver tumor were found in tree shrews of group A, namely 98–2 and 121–1, at their late stage of life (6.1 and 5.3 years of age respectively). Their other organs, including heart, lung, spleen, kidney, stomach and intestine, were basically normal by both gross and microscopic examinations. No tumor was found in the tree shrews of group B. The general information of the two cases of liver tumor is listed in Table 3.

**Table 3:** **General information of the two cases of liver tumor**

**Information**	**Animal**	
	**98-2**	**121-1**
Body weight (g)	142.0	117.7
liver weight (g)	13.6	21.2
Size and location of the liver tumor	Single nodule sized 2.5×2×2 cm, located at the bottom of the right posterior lobe, without capsule but with a visible boundary zone on its cross section.	Single nodule without obvious edge, sized about 3×3×2.5 cm, occupied and merged most parts of the left, middle and right posterior lobes. A cyst located on the surface of the tumor, sized 2.5×2×1.8 cm, containing yellow clear liquor.

**Figure 2:**
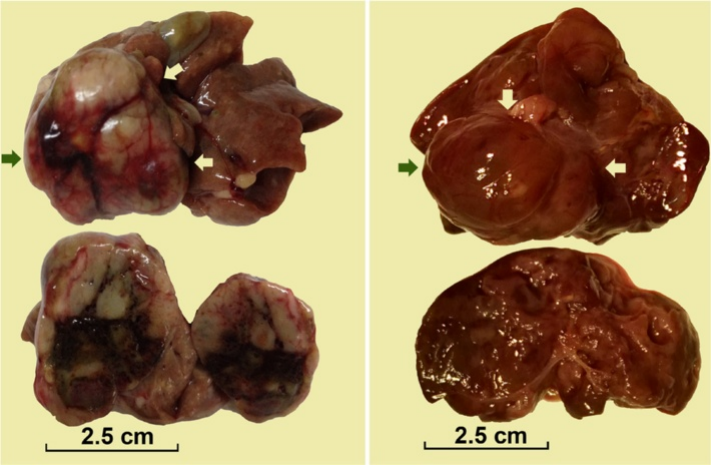
**Gross images of liver tumors.** The figures show the overall appearance and cross sections of the liver tumors developed in tree shrews 98–2 (left) and 121–1 (right) respectively. Generally, both tumors were solitary nodule and oval in shape, with uneven and mottled surface. The textures of the tumors were brittle. The cross sections of the nodules were nonuniform in color, mostly grayish white (lower left) or dark red (lower right). The three arrows in each picture point the edge of the tumor. The scale in each picture indicates tumor size.

The overall appearance and the cross-section’s view of the two liver tumors are shown in Figure 2. Generally, both tumors were single nodule and oval in shape, with uneven and mottled surface. The cross-sections of the nodules exhibited uneven color, mostly grayish white or dark red, and the parenchyma of the tumors were brittle.

**Figure 3:**
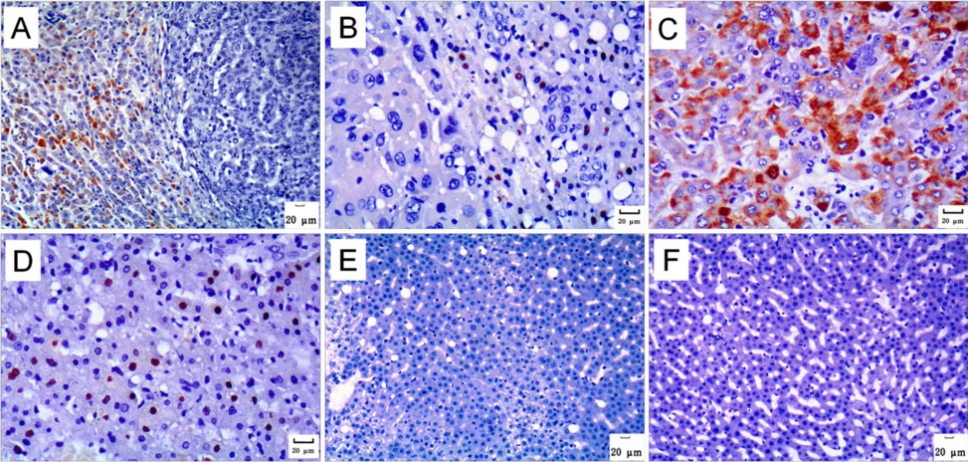
**HBsAg and HBcAg immunohistochemistry. A**. HBsAg in liver tumor and tumor-surrounding tissues. HBsAg-positive hepatocytes distributed sporadically or diffusely in the tumor-surrounding tissue (left area). On the contrary, HBsAg-positive cells were rarely seen inside the tumor (right area). Tumor and tumor-surrounding tissues of 98–2, immunohistochemical staining for HBsAg, 100×. **B**. HBcAg in liver tumor and tumor-surrounding tissues. HBcAg-positive hepatocytes distributed sporadically or diffusely in the tumor-surrounding tissue (right area). On the contrary, the HBcAg-positive cell was not observed inside the tumor (left area). Tumor and tumor-surrounding tissues of 121–1, immunohistochemical staining for HBcAg, 200×. **C**. HBsAg in biopsies collected before occurrence of liver tumor. HBsAg appeared in the cytoplasm of some hepatocytes. A liver biopsy specimen of 121–1 at the 221st week after inoculation, immunohistochemical staining for HBsAg, 200×. **D**. HBcAg in biopsies collected before occurrence of liver tumor. HBcAg appeared in the nucleus of some hepatocytes. A liver biopsy specimen of 121–1 at the 221st week after inoculation, immunohistochemical staining for HBcAg, 200×. **E**. HBsAg in the biopsies of animals in group B. No HBsAg-positive hepatocyte was found in the liver biopsies from animals in group B. A liver biopsy specimen of 108–4 at the 311th week after inoculation, immunohistochemical staining for HBsAg, 100×. **F**. HBcAg in the biopsies of animals in group B. No HBcAg-positive hepatocyte was found in the liver biopsies from animals in group B. A liver biopsy specimen of 108–4 at the 311th week after inoculation, immunohistochemical staining for HBcAg, 100×.

HBsAg and HBcAg Immunohistochemical staining on the two cases of liver tumor showed sporadically or diffusely distributed HBsAg- and HBcAg-positive hepatocytes in tumor-surrounding (peri-tumor) tissues, respectively, while fewer HBsAg-positive cells and none HBcAg-positive cells in tumor tissues (Figure 3A and B). Long-term observation on the liver biopsies from all the animals in group A reveals that, generally, HBsAg- and HBcAg-positive hepatocytes appeared early and increased slowly over time (Figure 3C and D). No HBsAg- or HBcAg-positive hepatocyte was found in any animals of group B at any time point during the years of observation (Figure 3E and F).

**Figure 4:**
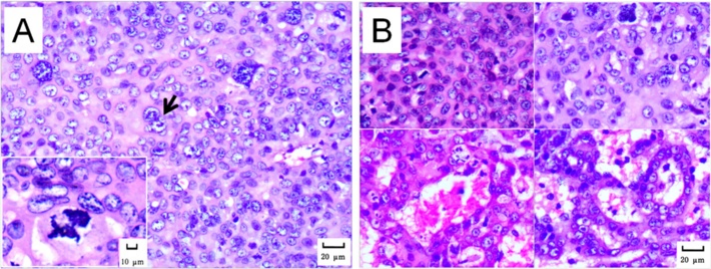
**Histological images of the liver tumors. A**. Neoplastic cells. The neoplastic cells showed marked cellular pleomorphism. Multinucleated cells (arrow) and abnormal mitoses (insert) were evident. Tumor tissue of 98–2, HE staining, 200× and 400× (insert) respectively. **B**. Morphological patterns. Three morphological patterns were found in tumors. The solid pattern was the tumor cells arranged in sheets with little intervening stroma and lack of sinusoids (two upper panels). The trabecular pattern was the tumor cells arranged in short and irregular cords (lower left panel). The pseudoglandular pattern was the tumor cells arranged as glandular structures (lower right panel). Tumor tissues of 98–2 (upper panels) and 121–1 (lower panels), HE staining, 200×.

Under microscope, the tumor cells showed typical hepatocyte-like features such as round, cuboidal or polygonal in shape. Some cells exhibited marked atypia and pleomorphism with enlarged nuclei and darker chromatin, as well as increased ratio of nucleus to cytoplasm. Numerous multinucleated cells presented, and mitotic figure and abnormal mitoses were frequently observed (Figure 4A). Some of the tumor cells showed transparent cytoplasm, which were proved to be rich in glycogen by periodic acid-Schiff (PAS) staining. The tumor cells arranged in solid, trabecular or pseudoglandular patterns (Figure 4B). Invasive growth, necrosis and hemorrhage presented in some areas of the tumors. The peri-tumor tissues exhibited chronic hepatitis changes, some with dysplastic nodules, but no significant fibrosis or cirrhosis was observed.

**Figure 5:**
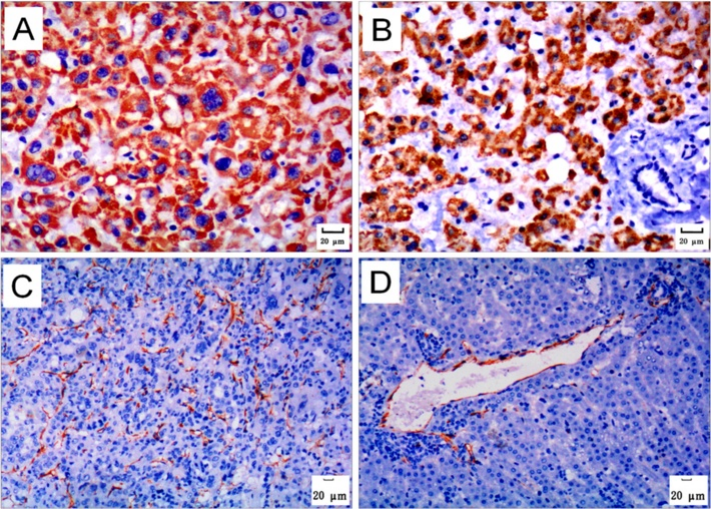
**HepPar-1 and CD31 immunohistochemistry. A**. HepPar-1 in the liver tumor tissue. The neoplastic cells showed diffusely HepPar-1 positive. A tumor tissue of 121–1, immunohistochemical staining for HepPar-1, 200×. **B**. HepPar-1 in the normal liver tissue. Normal hepatocytes generally showed HepPar-1 positive. A liver tissue of 131–3, immunohistochemical staining for HepPar-1, 200×. **C**. CD31 in the liver tumor tissue. CD31 was densely stained along the membrane of the sinusoidal endothelial cells. A tumor tissue of 121–1, immunohistochemical staining for CD31, 100×. **D**. CD31 in the normal liver tissue. Liver sinusoidal endothelial cells in the non-tumor tissue were CD31 negative, only blood vessels in portal tracts were positive. A liver tissue of 137–2, immunohistochemical staining for CD31, 100×.

Figure 5 shows part of the results of immunohistochemical study that helped to determine the nature of the two liver tumors. Strong positive HepPar-1 cells were observed in both tumor and normal liver tissues (Figure 5A and B). CD31-positive sinusoidal endothelial cells, arranged in special strip-like or branch-like patterns, were found in tumor tissues (Figure 5C). The hepatic sinusoidal endothelial cells in non-tumor tissues were generally CD31-negative, here only blood vessels in portal tracts and/or a fewer sinusoidal spaces near portal tracts were positive (Figure 5D).

All of the features mentioned above suggested that the two liver tumors were moderately to poorly differentiated HCC. Besides, as our pervious description [17], the liver biopsies collected from the two HCC-carried tree shrews before the appearance of HCC showed slowly aggravated changes of hepatitis, such as piecemeal necrosis and periportal inflammation.

### Progress of hepatocyte proliferation during HCC development identified by Ki67 immunohistochemistry

**Figure 6:**
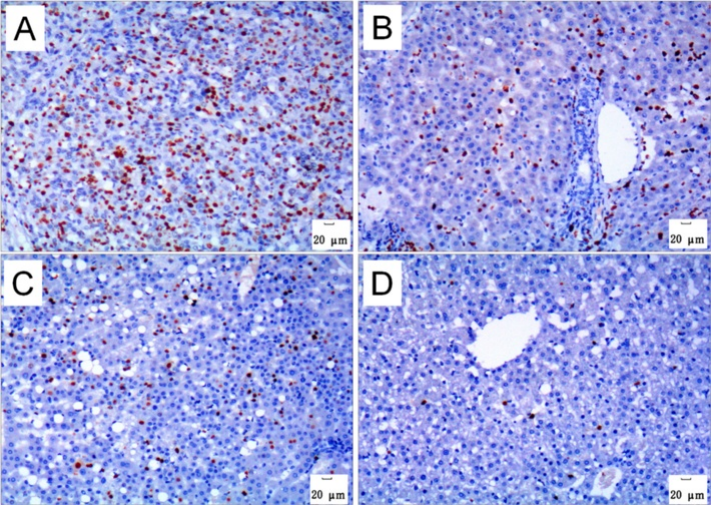
**Ki67 immunohistochemistry.** The density of Ki67-positive cells inside the live tumor **(A)** was much higher than that in the tumor-surrounding tissue **(B)**, the liver biopsies collected before occurrence of liver tumor **(C)** and the liver biopsies collected from animals in group B **(D)**. Immunohistochemical staining for Ki67, 100×.

As shown in Figure 6, the density of Ki67-positive cells in the HCC tissue was much higher than that in the compared tissues such as peri-HCC tissues, liver biopsies collected from the animals in group A prior to HCC occurrence (pre-HCC), and the liver biopsies collected from the animals in group B long after HBV-inoculation. The proportion (%) of Ki67-positive cells in HCC tissues was significantly higher than that in the liver biopsies of group B (Table 4).

**Table 4:** **Proportion (%) of Ki67-positive cells in HCCs and the comparable tissues**

**Tissue**	**Amount of samples**	**Mean ± SD (%)**	**P value (compare to HCC)**
HCC	2	21.70 ± 6.65	
Peri-HCC	2	10.00 ± 0.57	0.333
*Liver biopsies of group A	6	8.93 ± 6.02	0.143
*Liver biopsies of group B	9	5.27 ± 5.25	0.036

*Liver biopsies for Ki67 staining were collected around the 248th week after inoculation from the animals in group A and B, respectively.

## Discussion

The data presented above indicate that HCC can develop in the tree shrews with chronic HBV infection. Their HBV-related serological, hepatic histopathological and Immunohistochemical changes are similar to those of HBV-infected humans. These animals were nursed artificially in our lab from birth, without any other treatment except inoculation with HBV at their neonatal period and then periodical collection of blood sample and liver biopsy throughout their lifetime. As no HCC was found in the nine controls that were not chronically HBV-infected during several years of observation, as well as no spontaneous HCC in tree shrew has ever been found in our lab or reported by any others up to now, the conclusion might be drawn therefore, that the HCCs presented in this study were likely induced by HBV.

The histopathological changes observed in the two tree shrews’ HCC and peri-HCC tissues are similar to those observed commonly in human HCC. Furthermore, the immunohistochemical results of HepPar-1, CD31 and Ki67 suggest tree shrew’s HCC possesses certain phenotypes that are similar to that of human HCC. These markers had the supplementary diagnostic value for human HCC in clinical pathology. For examples, HepPar-1 is helpful in determining whether the tumor cells are hepatocyte-derived; CD31 is an angiogenesis-related marker for identifying cancer tissue from normal tissue, and Ki67 (PCNA) is a marker indicating cell proliferation [18-20]. Particularly, Ki67 was found positively related to HCC progression in human and mice [20,21], while our result of Ki67 staining showed the same tendency during the development of tree shrews’ HCC. Meanwhile, our result showed that the distributions of HBsAg- and HBcAg-positive hepatocytes were more often in peri–HCC tissues of tree shrews but much less in HCC tissues themselves, which are similar again to those observed commonly in human HBV-related HCC [22]. All of these evidences, as well as the history of HBV infection, consistently support that the two liver tumors of tree shrews are analogous to the HBV-related HCC in human being.

Even though all the tree shrews in this study were HBV-inoculated neonatally and then nursed artificially, their outcomes were notably various. For instance, except the two tree shrews in group A (97–1 and 122–1) died earlier, the remaining four in this group were observed for long time. While among these four, the two animals (98–2 and 121–1) that showed typical and pronounced serological changes, i.e., consistently positive of HBsAg, HBeAg and HBcAb, as well as elevated levels of HBV DNA, finally developed HCC in their late stage of life. While in the other two with relatively moderate serological changes, one (90–1) showed mild hepatic fibrosis and obvious dysplastic hepatocytes which is generally believed as precancerous change of HCC [17]; the last (123–3) is still survival, without histologically confirmed HCC yet, but suspected liver nodules were detected by B-ultrasound recently (data not shown). Whereas, the tree shrews in group B were not chronically infected after HBV inoculation, based on the long-term observation of their serum samples and liver biopsies. This phenomenon, similar to the situation observed in HBV-infected humans, indicates that the outcome after HBV infection is not only determined by HBV but also by some of host’s factors.

The present study proves preliminarily that HBV alone can induce HCC, but the carcinogenic ability of HBV is relatively weak, because it requires a much longer period of time to induce HCC when comparing to AFB1, another important etiologic factor for HCC in southern China and Africa. According to the reports from our lab and others [13,14,23], the period of time for AFB1 alone to induce HCC in tree shrews was much shorter: no more than 172 weeks. Besides, the HBV genotype B used in this study [16] might be partially responsible for the long induction time, since the HBV genotype C and sub-genotype Ce are reported to have higher risk for HCC [24,25]. Meanwhile, it is worthwhile to note that the tree shrew 121–1, one of the HCC-carriers in this study, was inoculated with serum from another tree shrew (90–1) who had been previously diagnosed as having chronic HBV infection. Compared with another HCC-carrier 98–1 who was inoculated with serum from a HBV-infected patient, the period of HCC development in tree shrew 121–1 was relatively shorter and the size of HCC was relatively bigger. This might imply that the HBV adapted in vivo of tree shrew own more potent to induce HCC in the same species. However, the conclusions on these points are limited by the small sample size of this study.

## Conclusions

The present study proves preliminarily that neonatal inoculation with HBV, consequently resulting in persistent HBV infection, is capable of inducing HCC in tree shrews. The tree shrew model could be a practical platform for studying the underlying mechanisms of HBV infection, as well as the related therapeutic measures.

## Materials and methods

### Animal experiment

The study protocol was approved by the Ethical Committee of Guangxi Tumor Hospital in accordance with the guidelines issued by Chinese government, which conforms to the criteria outlined in the “Guide for the Care and Use of Laboratory Animals” prepared by the National Academy of Sciences and published by the National Institutes of Health (NIH publication no. 86–23 revised 1985).

The tree shrews used in this study were descended from a population of wild tree shrews (Tupaia belangeri chinensis) originating from the Kunming Institute of Zoology, Chinese Academy of Science (Yunnan, China). They were reared artificially since birth, housed at the Laboratory Animal Center of Guangxi Medical University, and monitored by veterinarians.

All the tree shrews presented in this study were followed-up from an experiment described in our previous reports [16,17], which 46 neonatal tree shrews were inoculated with HBV-infected sera, and six tree shrews were then diagnosed as having chronic HBV infection since the presences of HBV-infection markers were longer than 48 weeks after inoculation. These six chronically HBV-infected tree shrews were grouped to A in this study, while group B recruited nine tree shrews that were confirmed as non-chronically HBV-infected by long-term observation after HBV inoculation and served as controls.

The methods of HBV inoculation and sample collection were described previously [16,17]. Briefly, newborn tree shrews were subcutaneously injected with HBV-infected serum (HBsAg-, HBeAg- and HBcAb-positive, HBV DNA ≥ 10^7^ IU/ml) twice, 300 ~ 500 μl/animal each time, on the first and third days after birth, respectively. The HBV inoculum was either from human HBV-carriers or from an infected tree shrew (90–1), all of which were HBV genotype B [16]. Serum samples and liver biopsies were collected periodically from each animal after inoculation. Serum samples were used for testing HBV-infection markers, while liver biopsies were used for HBV DNA, histopathological and immunohistochemical examinations.

### Detection of HBV serological markers and HBV DNA in serum and liver

All the methods of enzyme-linked immunoabsorbent assay (ELISA), time-resolved immunofluorescence analysis (TRFIA) and fluorescence quantitative polymerase chain reaction (FQ-PCR) for detecting HBV-infection markers were carried out by the Clinical Laboratory Center of Guangxi Tumor Hospital (Nanning, China). The operations were conducted by using validated procedures for clinical specimens, and according to the manufacturer’s instructions.

ELISA was applied to detect HBsAg, HBsAb, HBeAg, HBeAb and HBcAb in serum, by using an automatic processor ML-FAME (AusBio Company, Bonaduz, Switzerland) and the commercial kits from Kehua Bio-engineering Co. (Shanghai, China). The HBsAg-positive specimens were then further analyzed quantitatively by TRFIA, which was conducted on an automated immunoassay system Wallac AutoDELFIA (PerkinElmer Co., Turku, Finland) with the commercial kits from Xinbo Biotechnology Co. (Suzhou, China).

HBV DNA in serum and in liver biopsies was assessed quantitatively by FQ-PCR. This assay was performed on an analyzer Light Cycle® 480 II (Roche Diagnostics GmbH., Mannheim, Germany) with the commercial kit careHBV PCR ASSAY (QIAGEN Shenzhen Co., Ltd., Shenzhen, China). Before the FQ-PCR, liver DNA was extracted by using a QIAamp DNA Blood Mini Kit (Qiagen, Hilden, Germany) with a modified procedure as our previously reports [16,17]. The results of FQ-PCR were expressed as IU/ml in serum and as IU/μg liver DNA in liver. The threshold applied to classifying a tree shrew serum sample as positive was ≥5 × 10^2^ IU/ml, identical to the value recommended by the kit’s manufacturer for testing human samples. As no standard method and critical threshold for FQ-PCR detecting HBV DNA in liver tissues have been established so far, we employed the cut-off value as >1 × 10^4^ IU/μg liver DNA, by referring the results from detecting the controls from HBV-infected human and normal tree shrews.

### Histopathological and immunochemical observation on liver tissues

Hematoxylin and eosin (H&E) and immunohistochemical staining were performed on the paraffin-embedded liver biopsies, all in accordance with conventional procedures. The monoclonal antibodies of mouse anti-human HBsAg, HBcAg, HepPar-1, CD31 and Ki67 were purchased from Zhongshan Goldenbridge Biotech (Beijing, China). Ki67 stained slices were analyzed quantitatively, based on the reported method [20,21]. Briefly, 8–10 high-powered fields (×400) were randomly selected for measuring about 500 hepatocytes per specimen. The nuclei-positive hepatocytes were counted, and the results were expressed as proportion (%) of positive cells.

### Statistical analysis

Statistical analysis was conducted with SPSS 17.0 software (IBM, Chicago, USA). Wilcoxon Test was applied to compare the observation period of the two animal groups (Table 1) and the proportions of Ki67-positive hepatocytes in each comparable group (Table 4). The significance level was P <0.05.

## Abbreviations

ELISA: Enzyme-linked immunosorbent assay
FQ-PCR: Fluorescence quantitative polymerase chain reaction
HBV: Hepatitis B virus
HBsAg: HBV surface antigen
HBsAb: HBV surface antibody
HBeAg: HBV e antigen
HBeAb: HBV e antibody
HBcAg: HBV core antigen
HBcAb: HBV core antibody
HCC: Hepatocellular carcinoma
TRFIA: Time-resolved immunofluorescence analysis
